# Social Image Captioning: Exploring Visual Attention and User Attention

**DOI:** 10.3390/s18020646

**Published:** 2018-02-22

**Authors:** Leiquan Wang, Xiaoliang Chu, Weishan Zhang, Yiwei Wei, Weichen Sun, Chunlei Wu

**Affiliations:** 1 College of Computer & Communication Engineering, China University of Petroleum (East China), Qingdao 266555, China; richiewlq@gmail.com (L.W.); s16070788@s.upc.edu.cn (X.C.); zhanws@upc.edu.cn (W.Z.); z16070538@s.upc.edu.cn (Y.W.); 2 First Research Institute of the Ministry of Public Security of PRC, Beijing 100048, China; weichen.sun@hotmail.com; 3 School of Information and Communication Engineering, Beijing University of Posts and Telecommunications, Beijing 100876, China

**Keywords:** social image captioning, user-contributed tags, user attention, visual attention

## Abstract

Image captioning with a natural language has been an emerging trend. However, the social image, associated with a set of user-contributed tags, has been rarely investigated for a similar task. The user-contributed tags, which could reflect the user attention, have been neglected in conventional image captioning. Most existing image captioning models cannot be applied directly to social image captioning. In this work, a dual attention model is proposed for social image captioning by combining the visual attention and user attention simultaneously.Visual attention is used to compress a large mount of salient visual information, while user attention is applied to adjust the description of the social images with user-contributed tags. Experiments conducted on the Microsoft (MS) COCO dataset demonstrate the superiority of the proposed method of dual attention.

## 1. Introduction

Image caption generation is a hot topic in computer vision and machine learning. Rapid development and great progress have been made in this area with deep learning recently. We address the problem of generating captions for social images, which are usually associated with a set of user-contributed descriptors called tags [1,2,3]. Most existing works can not be used directly for social image captioning due to the accessible user-contributed tags.

**Figure 1:**
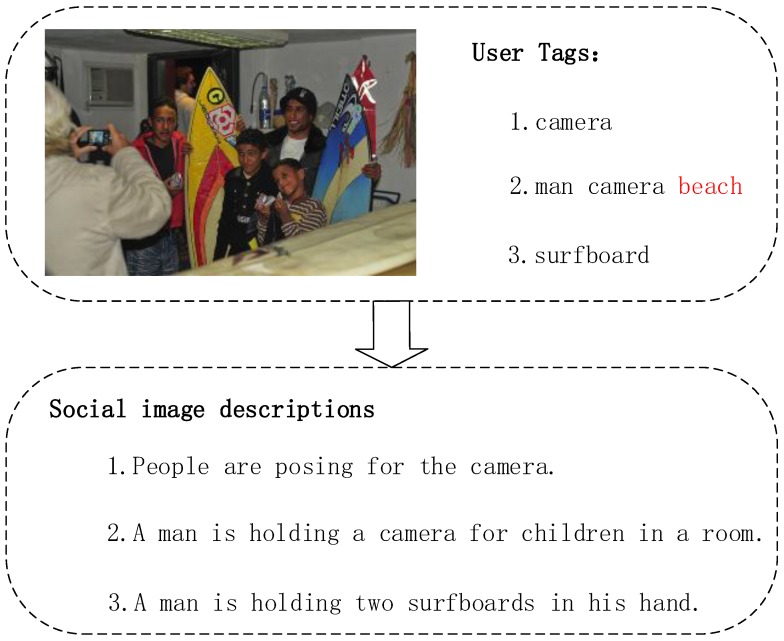
Social image captioning with user-contributed tags. Social image captioning demands personalized descriptions based on the diverse user attentions. In addition, incorrect user-contributed tags should be eliminated. The red word ‘beach’ can be considered as the noise of user tags referring to the image content.

The conventional image captioning task is to generate a general sentence for a given image. However, it is difficult to cover all the objects or incidents that appear in the image with a single sentence. As the old saying goes, “a picture is worth a thousand words”. This indicates that it may be insufficient for the current image captioning task. In fact, given an image, different people would generate various descriptions from their views. Conventional image captioning methods cannot obtain personalized user attention, which would be transferred into a corresponding description. Nowadays, most images are obtained from social media sites, such as Flickr, Twitter, and so on. These social images are usually associated with a set of user-contributed tags, which can express users’ attentiveness. The task of social image captioning is to generate descriptions of images with the help of available user tags. The social image captioning algorithm, according to what the users focus on, should consider the role of user tags on the basis of conventional image captioning models to generate corresponding descriptions (see Figure 1).

**Figure 2:**
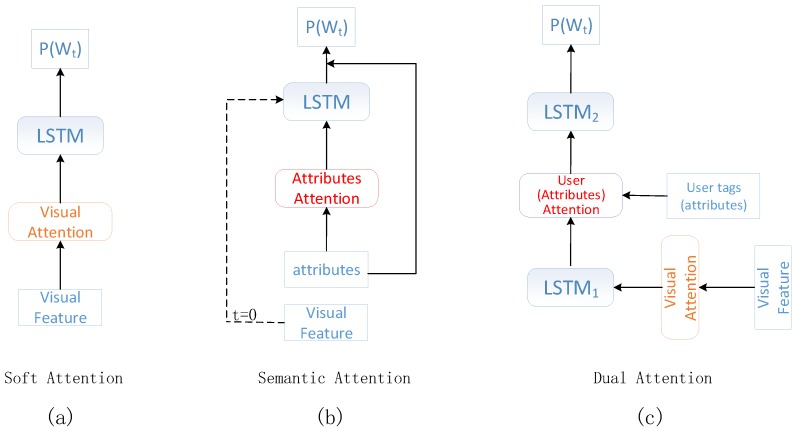
Comparisons with soft visual attention, semantic attention and proposed dual attention image captioning schemes. For the ease of comparisons, the role of user attention in the dual attention model can be regarded as attribute attention. (**a**) Only visual features are used to generate image captions in soft attention. (**b**) Attributes and visual features are used to generate image captions in semantic attention. However, visual features are used only at the first time step. (**c**) Both tags (attributes) and visual features are used to generate image captions in dual attention at every time step.

Great endeavors [4,5] have been made toward image captioning. However, most of them are not suitable for the specific task of social image captioning due to the existence of user tags. The attention-based image captioning method [4] is one of the most representative works. The attention mechanism is performed on the visual features, aiming to make an explicit correspondence between the image region and generated words. “Soft visual attention” [4] is proposed by Xu, where only visual features are used to generate image captions (see Figure 2a). However, this approach is not suitable for social image captioning. The information of user tags cannot be utilized directly in “soft attention” for social image captioning. The attribute-based image captioning method is another popular way of image captioning. Precise attributes are detected as the high-level semantic information to generate image captions. “Semantic attention” [5] is proposed by You to make full use of attributes to enhance image captioning (see Figure 2b). In such a situation, user tags can be incorporated into “semantic attention” by conducting a direct substitution with attributes. However, the visual features are not fully utilized in “semantic attention”, which overly depends on the quality of attributes. In the majority of cases, user-contributed tags are not always accurate, and much noise may be mixed in. The influence of noise is not well considered in the attribute-based image captioning methods. A qualified social image caption generator should emphasize the role of accurate tags, meanwhile eliminating the effects of noisy tags. How to balance the visual content and the user tags should be well investigated for social image captioning.

In this paper, we propose a novel dual attention model (DAM) to explore the social image captioning based on visual attention and user attention (see Figure 2c). Visual attention is used to generate visual descriptions of social images, while user attention is used to amend the deviation of visual descriptions to generate personalized descriptions that conform to users’ attentiveness.

To summarize, the main contributions of this paper are as follows:

- Social image captioning is considered to generate diverse descriptions with corresponding user tags. User attention is proposed to address the different effects of generated visual descriptions and user tags, which lead to a personalized social image caption.
- A dual attention model is also proposed for social image captioning to combine the visual attention and user attention simultaneously. In this situation, generated descriptions maintain accuracy and diversity.

## 2. Related Work

The attention-based V2Lmodel improves the accuracy of image captioning. When generating corresponding words, these attention-based V2L models [6,7,8] incorporate an attention mechanism that imitates the human ability to obtain the information of images [4,9]. Zhou et al. [10] proposed a text-conditional attention mechanism, which allows the caption generator to focus on certain image features based on previously-generated texts. Liu et al. [11] and Kulkarni et al. [12] introduced a sequential attention layer that considers the encoding of hidden states to generate each word. Xiong et al. [13] proposed an adaptive attention model, which is able to decide when and where to attend to the image. Park et al. [14] proposed a context sequence model, where the attention mechanism is performed on the fused image features, user context and generated words. Chen et al. [15] put forward a spatial and channel-wise attention mechanism. It learns the relationship between visual features and hidden states. However, these attention-based image caption methods cannot be used directly for social image captioning due to the additional user-contributed tags. The proposed DAM is also built on the attention mechanism. Besides, user attention is considered to generate social image captions by incorporating user-contributed tags.

The attribute-based V2L model utilizes high-level concepts or attributes [16] and then injects them into a decoder with semantic attention to enhance image captioning. Kiros et al. [17] proposed a multimodal log-bilinear neural language model with attributes to generate captions for an image. Vinyals et al. [18] introduced an end-to-end neural network, which utilized LSTM to generate image captions. Recently, You et al. [5] utilized visual attributes [19,20] as semantic attention to enhance image captioning. At present, in most image caption models, each word is generated individually according to the words’ sequence in the caption. However, for the human, they are more likely to determine firstly what the objects are in the image, what the relationship is between objects and then describe every object with their remarkable characteristics. Wang et al. [21] put forward a coarse-to-fine method, which described the image caption in two parts, a main clause (skeleton sentence) and a variety of features (attributes) of the object. Ren et al. [22] proposed a novel decision-making framework that uses both the “policy network” and “value network” to generate descriptions. The policy network plays a local guidance role, while the value network plays a global and forward-thinking guidance role. However, the quality of attributes directly determined the performance of captions generated by the traditional attribute-based methods. The noisy user-contributed tags have to be considered carefully to be excluded from the generated captions. In this paper, the problem has been solved by simultaneously combining visual attention and user attention, which are incorporated into two parallel LSTMs.

## 3. Proposed Method

### 3.1. Preliminaries

The encoder-decoder framework [23,24] is a popular architecture in the field of image captioning. The encoder converts the input sequence into a fixed length vector, and the decoder converts the previously-generated fixed vector into the output sequence. The image is commonly represented by a CNN feature vector as the encoder, and the decoder part is usually modeled with recurrent neural networks (RNN).

RNN is a neural network adding extra feedback connections to feed-forward networks, so as to work with sequences. The update of the network [7,24] relies on the input and the previous hidden state. The hidden states $\left(h_{1},h_{2},\ldots ,h_{m}\right)$ of RNN are computed based on the recurrence of the following form given an input sequence $\left(a_{1},a_{2},\ldots a_{m}\right)$.

(1) $$h_{t}=\varphi \left(W_{h}a_{t}+U_{h}h_{t-1}+b_{h}\right)$$

where weight matrices *W*, *U* and bias *b* are parameters to be learned and φ() is an element-wise activation function.

As illustrated in previous works, the long short-term memory (LSTM) achieves a better performance than vanilla RNN in image captioning. Compared with RNN, LSTM not only computes the hidden states, but also maintains a cell state to account for relevant signals that have been observed. They could modulate information to the cell state by gates.

Given an input sequence, the hidden states and cell states are computed by an LSTM unit via repeated application of the following equations:

(2) $$i_{t}=\sigma \left(W_{i}a_{t}+U_{i}h_{t-1}+b_{i}\right)$$

(3) $$f_{t}=\sigma \left(W_{f}a_{t}+U_{f}h_{t-1}+b_{f}\right)$$

(4) $$o_{t}=\sigma \left(W_{o}a_{t}+U_{o}h_{t-1}+b_{o}\right)$$

(5) $$g_{t}=\varphi \left(W_{g}a_{t}+U_{g}h_{t-1}+b_{g}\right)$$

(6) $$c_{t}=f_{t}⊙c_{t-1}+i_{t}⊙g_{t}$$

(7) $$h_{t}=o_{t}⊙c_{t}$$

where σ() is the sigmoid function, φ() represents the activation function and ⊙ denotes the element-wise multiplication of two vectors.

### 3.2. Dual Attention Model Architecture

Given a social image s∈S and a set of user tags $T_{i}\left(i=1,2\ldots .,m\right)$, the task of social image captioning is to generate *m* captions $c_{i}\left(i=1,2\ldots .,m\right)$ according to the corresponding user tags $T_{i}$. For simplicity, we reformulate the problem to utilize social image and its associated user-contributed noisy tags (s,T) to generate a personalized caption *c*.

The convolutional neural network (CNN) is firstly used to extract a global visual feature for image *s* denoted by $v=\{v_{1},v_{2},\ldots v_{L}\}$, which represents the features extracted at different image locations. In addition, we get a list of user tags $T\in R^{n\times |D|}$ that can reflect users’ attentiveness. Here, $T=\{T_{1},T_{2},\ldots T_{n}\}$, *n* is the length of tags, and each tag (as well as generated word) corresponds to an entry in dictionary *D*.

**Figure 3:**
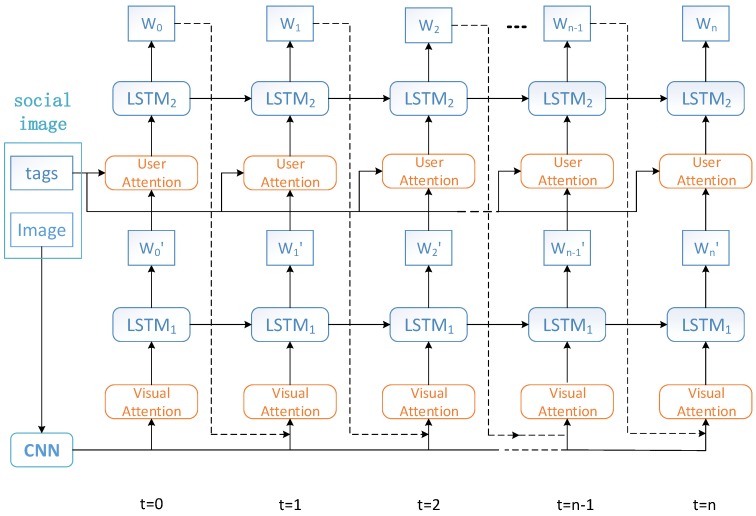
The architecture of the proposed dual attention model. The image feature is fed into the visual attention model to generate the visual word $W^{{}^{\prime }}$ based on the hidden state $h_{1}$ of $LSTM_{1}$. $W^{{}^{\prime }}$ and tags are then combined to generate word *W* by user attention based on the hidden state $h_{2}$ of $LSTM_{2}$. The user attention modal is exploited to emphasize the users’ attentiveness and to eliminate the influence of noisy tags.

All visual features [25], processed by the visual attention model, are fed into the first long short-term memory layer ($LSTM_{1}$) to generate word $W_{t}^{{}^{\prime }}$ at time *t*. The generated word $W_{t}^{{}^{\prime }}$ and user tags are combined as the new input for the user attention model. The user attention adds an additional consideration of tags on the basis of the original image, which is then passed to the second LSTM layer ($LSTM_{2}$) to generate word $W_{t}$. The architecture of the dual attention model is illustrated in Figure 3. Different from previous image captioning methods, the caption generated by our method is consistent with the users’ attentiveness; meanwhile, it corrects deviation caused by the noisy user tags. The dual attention model can be regarded as a coordination of image content and users’ attentiveness. Specifically, the main workflow is governed by the following equations:

(8) $$V_{att}=\left\{\begin{array}{cc} f_{vatt}\left(v\right), & t\;=\;0\\ f_{vatt}\left(v,W_{t-1}\right), & t\;>\;0 \end{array}\right.$$

(9) $$W_{t}^{{}^{\prime }}=LSTM_{1}\left(V_{att},h_{t-1}^{1}\right)$$

(10) $$E_{t}=f_{uatt}\left(W_{t}^{{}^{\prime }},T\right)$$

(11) $$W_{t}=LSTM_{2}\left(E_{t},h_{t-1}^{2}\right)$$

Here, $f_{vatt}$ is applied to attend to image feature *v*. $V_{att}$ represents the image feature that is processed by the visual attention model. $f_{uatt}$ is used to attend to user attention (*T* and $W_{t}^{{}^{\prime }}$). For conciseness, we omit all the bias terms of linear transformations in this paper. The visual information $W_{t}^{{}^{\prime }}$ and user tags *T* are combined by Equation (10) to remove the noise of social images. Equations (8)–(11) are recursively applied, through which the attended visual feature and tags are fed back to the hidden states $h_{t}^{1}$ and $h_{t}^{2}$ respectively.

### 3.3. Visual Attention

The soft attention [8] mechanism is used in the visual attention part to deal with visual information. When t>0, based on the previous predicted word $W_{t-1}$, the visual attention model assigns a score $\alpha_{t}^{i}$. The weight $\alpha_{t}^{i}$ is computed by using a multilayer perception conditioned on the previous word $W_{t-1}$. The soft version of this attention $G_{att}$ was introduced by [8]. The details are as follows:

(12) $$K_{t}^{i}=\left\{\begin{array}{cc} G_{att}\left(v\right), & t=0\\ G_{att}\left(v,W_{t-1}\right), & t>0 \end{array}\right.$$

(13) $$\alpha_{t}^{i}=\frac{exp(K_{t}^{i})}{\sum_{1}^{L}exp(K_{t}^{i})}$$

Once the weights (which sum to one) are computed, the $V_{att}$ could be computed by:

(14) $$V_{att}=\sum_{1}^{L}V_{i}\alpha_{t}^{i}$$

The $V_{att}$ and $h_{t-1}^{1}$ will be fed into $LSTM_{1}$ [26] to generate visual word $W_{t}^{{}^{\prime }}$. The visual attention model is a mid-level layer, which provides an independent overview of the social image no matter what the user focus is.

### 3.4. User Attention

The user-contributed tags may reflect users’ preference, which should be considered carefully in the social image captioning. At the same time, the user-contributed tags are usually overabundant with noisy and misleading information. For these reasons, the user attention model is proposed to address the above issues. $W_{t}^{{}^{\prime }}$ and tags *T* were merged into $Q=\{T,W_{t}^{{}^{\prime }}\}$. For t>0, a score $\beta_{t}^{i}\left(i=1,2,\ldots ,n+1\right)$ is assigned to each word in *Q* based on its relevance with the previous predicted word $W_{t-1}$ and the current hidden state $h_{t}^{2}$ of $LSTM_{2}$. Since $W_{t}^{{}^{\prime }}$ and *T* correspond to an entry in dictionary *D*, they can be encoded with one-hot representations. The details of the user attention model are as follows:

(15) $$e_{t}^{i}=G_{att}\left(EQ\right)$$

(16) $$\beta_{t}^{i}=\frac{exp(e_{t}^{i})}{\sum_{1}^{n+1}exp(e_{t}^{i})}$$

where Equation (15) is used to compute the weights of *Q*, and $G_{att}$ has the same function as Equation (12). The matrix *E* contains parameters for dictionary *D* with a reasonable vocabulary size. Equation (16) is used to normalize $\beta_{t}^{i}$, and n+1 is the length of *Q*.

(17) $$Z_{att}=\sum_{1}^{n+1}Q_{i}\beta_{i}^{t}$$

Finally, the hidden state $h_{t-1}^{2}$ and the matrix $Z_{att}\in R^{\left|D\right|}$ that involves the information of user attention are fed into $LSTM_{2}$ to predict the word $W_{t}$, which involves the user attention.

### 3.5. Combination of Visual and User Attentions

The framework of [5] injected visual features and attributes into RNN, then fused together through a feedback loop. This method only uses visual features at t=0, which ignores the role of visual information in the LSTM. What’ is more, this leads to an over-reliance on the quality of the attributes. Once the attributes are noisy, the performance of the algorithm [5] drops sharply. Thus, a pair of parallel LSTMs is pushed forward to balance the visual and semantic information. Firstly, image features are fed into $LSTM_{1}$ to decode visual word $W^{{}^{\prime }}$, which can reflect the objective visual information, then $W^{{}^{\prime }}$ and tag $T_{i}\left(i=1,2\ldots .,m\right)$ are encoded into $LSTM_{2}$ for further refinement of visual information feedback. For example, visual attention generated the word “animals” by $LSTM_{1}$, and the word “dog” was addressed in the user tags. $LSTM_{2}$ is able to further translate the “animals” into “dog” for feedback and enhancement of visual information. In other words, the proposed method could maintain a balance between the image content and user tags.

## 4. Experimental Results

### 4.1. Datasets and Evaluation Metrics

Experiments were conducted on MS COCO (Microsoft, Redmond, Washington, DC, USA) [27] to evaluate the performances of the proposed model. MS COCO is a challenging image captioning dataset, which contains 82,783, 40,504 and 40,775 images for training, validation and testing, respectively. Each image has five human-annotated captions. To compare with previous methods, we follow the split from previous work [4,5]. The image features are extracted by VGG-19 [28]. BLEU (bilingual evaluation understudy) [29], METEOR (metric for machine translation evaluation) [30] and CIDEr (Consensus-based Image Description Evaluation) [31] are adopted as the evaluation metrics. The Microsoft COCO evaluation tool [27] is utilized to compute the metric scores. For all three metrics, higher scores indicate that the generated captions are considered to be closer to the annotated captions created by humans.

The user tags of social images are key components of our model. As there is no ready-made dataset for social image captioning, two kinds of experiment schemes are designed on MS COCO as workarounds to compare fairly with the other related methods. Visual attributes and man-made user tags are respectively applied in the following subsections to validate the effectiveness of the proposed dual attention model (DAM).

### 4.2. Overall Comparisons by Using Visual Attributes

In this part, visual attributes are used for the first experiment scheme. The proposed DAM are compared with several typical image captioning methods. The methods are as follows:

- Guidance LSTM (gLSTM) [26] took the three different kinds of semantic information to guide the word generation in each time step. The guidance includes retrieval-based guidance (ret-gLSTM), semantic embedding guidance (emb-gLSTM) and image guidance (img-gLSTM).
- Soft attention [4] put forward the spatial attention mechanism that performed on the visual features. Different weights were assigned to the corresponding regions of the feature map to represent context information. The context information was then input to the encoder-decoder framework.
- Semantic attention [5] injected the attribute attention and visual features (t=0) into the LSTM layer.
- Attribute-based image captioning with CNN and LSTM (Att-CNN + LSTM) [32] used the trained model to predict the multiple attributes as high-level semantic information of the image and incorporated them into the CNN-RNN approach.
- Boosting image captioning with attributes (BIC + Att) [33] constructed variants of architectures by feeding image representations and attributes into RNNs in different ways to explore the correlation between them.

Exploiting the attributes of images [5,33] in advance is a recent popular way for image captioning. To be fair, visual attributes are also detected as special “user tags” in DAM, which is called DAM (attributes) here. Following [34], multiple instance learning is used to train the visual attribute detectors for words that commonly occur in captions. At last, four attributes are selected for each image to generate captions. The results are shown in Table 1. The proposed DAM (attributes) achieves the best performance among all methods, which validates the effectiveness of DAM. In addition, the methods with attributes [5,32,33] perform better than the other methods [4,13,26] in image captioning. Though the attributes are detected directly from the image, they can also be regarded as a kind of pre-knowledge for image captioning. From one side, the phenomenon illustrates that user tags can also be treated as another kind of pre-knowledge to generate accurate and personalized image descriptions. What is more, DAM (attributes) is further compared with “semantic attention” [5] in detail. Both use attributes and attention mechanism for image captioning. However, DAM (attributes) is a dual temporal architecture (two different LSTMs) with visual attention and user attention, while “semantic attention” uses a single LSTM layer. The user attention can be considered as a refinement of visual attention, which decreases the ambiguity generated by the first temporal layer.

### 4.3. Overall Comparison by Using Man-Made User Tags

In this subsection, man-made user tags are utilized as the second experiment scheme to further validate the effectiveness of DAM. Due to the lack of user tags on COCO, user tags were extracted from each description in advance. In each description, we randomly extracted 1–3 keywords (remove the prepositions and pronouns) as user tags to reflect the users’ attentiveness. That is, if an image with five descriptions, five corresponding groups of words could be extracted as user tags. To imitate “real social image” conditions, 7% noise (words from other images) was randomly added as user tags in the extraction process.

For fair comparisons, the classical attribute-based image captioning algorithms [5,32,33] are implemented by substituting the visual attributes with the man-made user tags. The same user tags are applied for all methods listed in Table 2. As shown in Table 2, DAM (tags) also achieves the best performance when using the man-made user tags. Due to the noisy characteristics of user-contributed tags, it may lead to a worse performance than using visual attributes. However, the proposed DAM (tags) performs better than the other attributed-based image captioning methods. From another side, the phenomenon illustrates that the proposed method has the advantage of noise resistance. In contrast, “semantic attention” [5] does not perform well when replacing attributes with noisy tags. It demonstrates that “semantic attention” does not apply to the description of social images with noise. As shown in Figure 2b, “semantic attention” depends much on the user tags (detected attributes), where visual features are not well utilized at each time step. On the contrary, the proposed DAM takes full advantage of user tags (or attributes) and visual features at each time step. The generated descriptions by DAM are still in keeping with the image content.

Generated descriptions should be in accordance with their corresponding user tags. Rather than being measured with all five ground-truth descriptions, a generated social image description should be measured with its corresponding ground-truth, from whence the user tags are extracted. However, the Microsoft COCO evaluation tool [27] computes a generated caption with five annotated ground-truths and preserves the highest score among the five comparisons. Based on the above consideration, the Microsoft COCO evaluation tool is modified for further comparisons. The results are reported in Table 3. The DAM (tags) also outperforms the other methods. A conclusion drawn from Table 2 and Table 3 is that DAM has the capability to generate accurate and diverse social image descriptions.

### 4.4. The Influence of Noise on the Dual Attention Model

**Figure 4:**
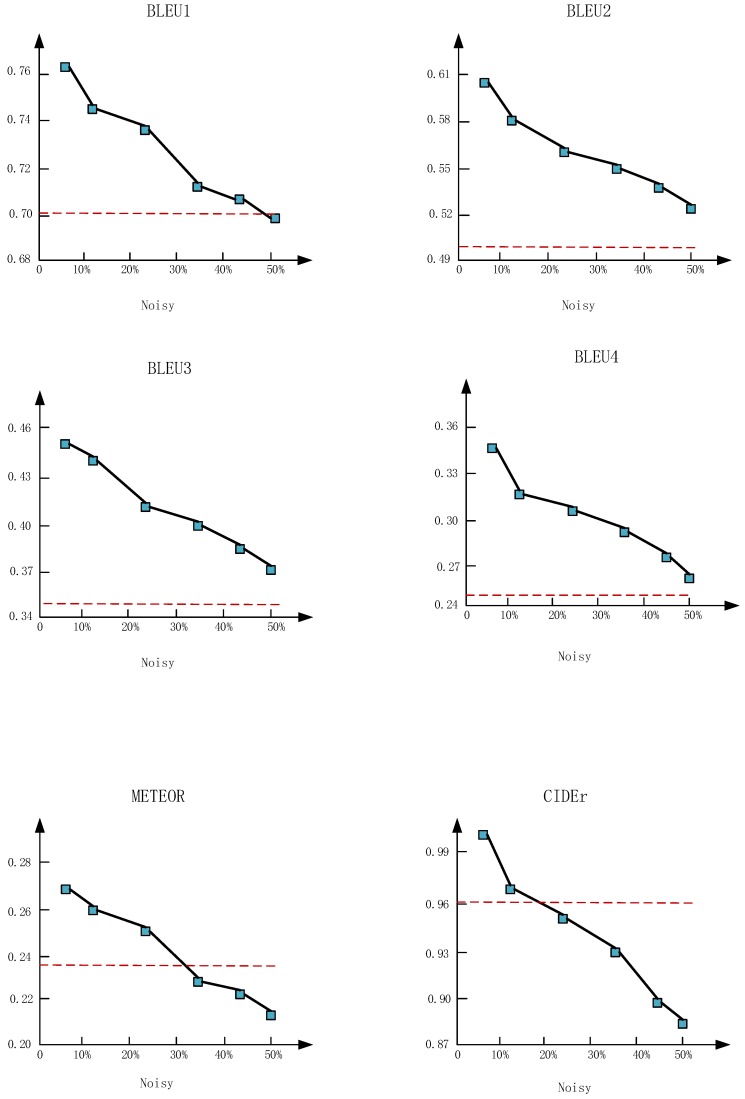
Variations of the performance with the increased noisy tags. The red dashes represent the results of the soft attention model [4].

To further investigate the influence of noise on DAM, different proportions of noise (7%, 10%, 25%, 33%, 43%, 50%) are added to user tags. Figure 4 shows the results with varied proportions of noise in the extracted user tags. The red dashes represent the results of “soft attention” [4] with merely visual features. Overall, the proposed DAM outperforms “soft attention” [4] on most metrics, even with a large proportion of noise in user tags. The DAM has the advantage of generating personalized captions while at the same time maintaining high performances. As the noise increased, the performance of DAM declined. However, the performance of DAM is also higher than the soft attention [4] when adding 43% noise. The results show that the proposed method is insensitive to noise, and the user tags have important effects on the image caption.

### 4.5. Qualitative Analysis

**Figure 5:**
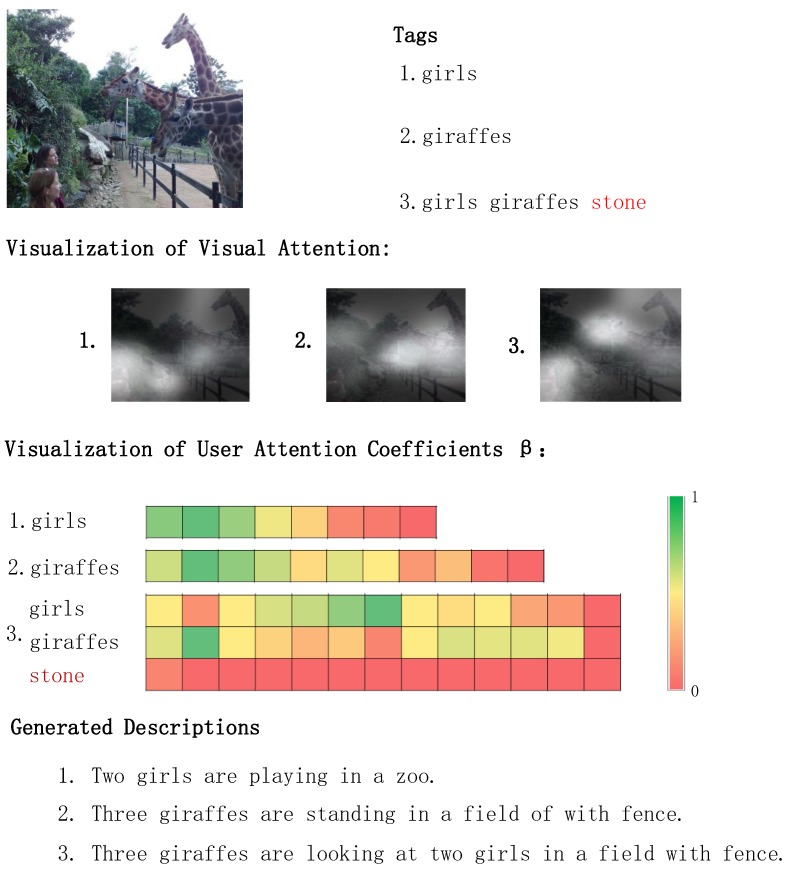
The visualization of visual attention and user attention weights β. The red word “stone” is the noise of the user tags. In the visualization of user attention, each grid represents the weight β of the tag when generating each word in the description.

In order to enhance the comprehension of the proposed model, the user attention was visualized in the process of social image captioning. As shown in Figure 5, different tags were applied to generate social image descriptions. The word “stone” is the man-made noise in Tag 3. The result of Description 3 proves the robustness of the proposed model, which has the capability of noise resistance. Even though noisy tags are provided, DAM could correct the deviation in terms of the real content of social images. If the tag is “giraffes”, the user attention model will adjust the visual attention to attend to the giraffes in the picture (see Description 2). The same situation can also be found in Description 1. The above examples show that the proposed model is able to adaptively select words from tags and integrate them well with the content of the image to generate personalized descriptions.

## 5. Conclusions

In this work, we proposed a novel method for social image captioning, which achieves good performances across the standard benchmarks. Different from previous work, the proposed DAM takes into account the user attention by using user-contributed tags of social images. The core of the proposed method lies in optimizing user attention to adaptively fuse global and local information for diverse descriptions. DAM is a kind of serial architecture for visual attention and user attention. The computational efficiency is of concern. Other architectures (such as parallel architectures) should be developed to further balance the visual content and user tags. For the next steps, we plan to experiment with user tags and visual attributes with diverse representations, as well as to explore new architectures for the dual attention mechanism.

## Figures and Tables

**Table 1:** Comparisons with recent image captioning methods. “*” stands for the attributes used in the method. gLSTM, guidance LSTM; DAM, dual attention model.

Method	MS-COCO					
	B-1	B-2	B-3	B-4	METEOR	CIDEr
gLSTM [26]	0.67	0.491	0.358	0.264	0.227	0.812
Soft Attention [4]	0.707	0.492	0.344	0.243	0.239	-
Semantic Attention * [5]	0.709	0.537	0.402	0.304	0.243	-
Att-CNN + LSTM * [32]	0.74	0.56	0.42	0.31	0.26	0.94
BIC+ Att * [33]	0.73	0.565	0.429	0.325	0.251	0.986
DAM (Attributes) *	0.738	0.570	0.432	0.327	0.258	0.991

**Table 2:** Comparisons with attribute-based image captioning methods. All methods apply man-made user tags for fair comparisons.

Method	MS-COCO					
	B-1	B-2	B-3	B-4	METEOR	CIDEr
Semantic Attention	0.710	0.540	0.401	0.298	0.261	-
Att-CNN + LSTM	0.689	0.524	0.387	0.285	0.249	0.883
BIC + Att	0.696	0.526	0.386	0.285	0.248	0.871
DAM (Tags)	0.76	0.597	0.452	0.342	0.261	1.051

**Table 3:** Comparisons by using man-made user tags with the modified evaluation tool.

Method	MS-COCO					
	B-1	B-2	B-3	B-4	METEOR	CIDEr
Semantic Attention	0.512	0.364	0.264	0.192	0.236	1.967
Att-CNN + LSTM	0.490	0.344	0.249	0.183	0.220	1.884
BIC + Att	0.485	0.343	0.251	0.188	0.219	1.090
DAM (Tags)	0.544	0.400	0.296	0.221	0.258	2.293
